# Evaluation of 10 SLE susceptibility loci in Asian populations, which were initially identified in European populations

**DOI:** 10.1038/srep41399

**Published:** 2017-01-27

**Authors:** Yue-miao Zhang, Xu-jie Zhou, Swapan K. Nath, Celi Sun, Ming-hui Zhao, Hong Zhang

**Affiliations:** 1 Renal Division, Peking University First Hospital, Peking University Institute of Nephrology, Key Laboratory of Renal Disease, Ministry of Health of China, Key Laboratory of Chronic Kidney Disease Prevention and Treatment (Peking University), Ministry of Education, Beijing, People’s Republic of China; 2 Arthritis and Clinical Immunology Research Program, Oklahoma Medical Research Foundation, Oklahoma City, Oklahoma, USA

## Abstract

Ten novel loci have been found to be associated with systemic lupus erythematosus (SLE) susceptibility by a recent genome-wide association study conducted in Europeans. To test their disease associations and genetic similarities/differences in Asians and Europeans, we genotyped the 10 novel single nucleotide polymorphisms (SNPs) and performed an association study. A Chinese cohort from Northern China was recruited as the discovery population, and three East Asian cohorts were included for independent replication. The 10 SNPs were genotyped using TaqMan allele discrimination assays. To prioritize the associated SNPs, different layers of the public functional data were integrated. Among the 10 SNPs, rs564799 in *IL12A* was shared in both ethnicities (*P*_adjust_ = 5.91 × 10^−4^; odds ratio = 1.22, 1.10–1.35). We also confirmed the reported polymorphism rs7726414 in *TCF7* in the current study (*P*_adjust_ = 4.12 × 10^−8^; odds ratio = 1.46, 1.28–1.66). The directions and magnitudes of the allelic effects for most of the 10 SNPs were comparable between Europeans and Asians. However, higher risk allele frequencies and population-attributable risk percentages were observed in Asians than in Europeans. We also identified the most likely functional SNPs at each locus. In conclusion, both genetic similarities and differences across ethnicities have been observed, providing further evidence for a genetic basis of the high incidence of SLE in Asian ancestry.

Systemic lupus erythematosus (SLE) is a chronic autoimmune disease with complex genetic etiology that can affect the majority of organs and tissues. The strong familial aggregation and high disease concordance in monozygotic twins (24–56%) have suggested a genetic component to SLE predisposition^1^. To date, based on hypothesis-free genome-wide association studies (GWAS), 62 SLE susceptibility loci have been identified in single ancestries^2^, providing important clues for understanding the genetic architecture of SLE patients.

However, the incidence of SLE varies across different populations, with a markedly higher prevalence in Asians than in Europeans^3^,^4^, suggesting genetic heterogeneity across populations. Although it was suggested that most of the novel SLE risk loci identified by GWAS was shared between Asians and Europeans^2^,^5^, a trans-ancestral comparison study would be helpful for uncovering genetic heterogeneity and detecting novel susceptibility loci in different populations^6^. Recently, 10 novel SNPs have been identified by a GWAS of SLE in European populations^7^. Among them, rs7726414 in *TCF7* has also been reported to be associated with Asian SLE patients^8^. Thus, in this study, by genotyping the 10 novel SNPs without selection, we initially tended to explore the genetic similarities/differences in Asians and Europeans by comparing the allelic associations, odds ratios (ORs), risk allele frequencies (RAFs), and population-attributable risk percentages (PARPs) of these novel risk alleles.

In addition to, considering that most of the SLE-associated variants are located in non-coding regions of the genome, the public Encyclopedia of DNA Elements (ENCODE) databases and expression quantitative trait loci (eQTL) mapping could provide us a novel perspective on interpreting functional single nucleotide polymorphisms (SNPs) with regulatory effects^9^. Thus, to prioritize the plausible functional SNPs and genes of the associated SNPs in this study, we integrated different layers of functional data, including DNase I hypersensitivity (DHS) analysis results, DNase I footprints, chromatin immunoprecipitation followed by sequencing (ChIP-seq) data, and eQTL mapping results according to the previous report^10^.

## Results

### Allelic association analyses

After quality control, 493 cases and 628 controls were included in the analysis. All 10 SNPs were in Hardy-Weinberg equilibrium in patients and controls (*P* > 0.05). As shown in Tables 1, three SNPs, including rs6740462 in *SPRED2*, rs564799 in *IL12A*, and rs2286672 in *PLD2* have been significantly detected (with *P* values ranging from 3.51 × 10^−2^ to 9.36 × 10^−5^). The variant rs7726414 in *TCF7* showed marginal significance in the discovery population (*P* = 5.37 × 10^−2^), while no solid evidence of associations was observed for the others (with *P* values of 0.16–0.77). For replication, genotype data for the 4 associated SNPs were then extracted from our previous study on East Asians, including Korean, Han Chinese and Malaysian Chinese^8^. Consistent associations of rs564799 in *IL12A* and rs7726414 in *TCF7* have been observed, and the significances were enhanced by meta-analysis. Interestingly, the association between these two SNPs and SLE remained significant after multiple corrections (*P* values were 5.91 × 10^−4^ and 4.12 × 10^−8^, respectively, using the Bonferroni method on 4 SNPs) (Table 2). The effects of the two associated alleles were in the same direction (either risk or protective factors for SLE) as reported in Europeans.

Detection powers for 10 SNPs in the Chinese Han population, assuming the odds ratios (ORs) in the published GWAS, are 55.1%, 61.6%, 71.7%, 75.8%, 80.9%, 92.6%, 95.1%, 95.7%, 96.8%, and 98.3% for rs9652601, rs887369, rs4902562, rs10774625, rs3768792, rs2286672, rs6740462, rs7726414, rs564799, and rs3794060, respectively. For the replicated 4 SNPs, rs7726414, rs564799, rs2286672, and rs6740462, genetic powers of the combined set of 2978 SLE cases and 4575 controls were 95.7%, 96.8%, 97.7%, and 99.5%. Thus, the un-replicated SNPs may be due to sample heterogeneity or limited detecting power (lower MAFs compared to Europeans).

### Comparisons of risk allele frequencies, effect sizes and risk across cohorts

As shown in Table 3, the risk allele frequencies (RAFs) of all 10 SNPs in the controls were significantly higher in Asians than in Europeans, with *P* values ranging from 1.93 × 10^−266^ to 4.54 × 10^−2^. Especially for rs4902562 in *RAD51B* and rs2286672 in *PLD2*, the minor alleles in Europeans were the major alleles in Asians. Consistently with the clear differences in RAFs between Asians and Europeans, the PARPs were higher for most of the 10 SNPs in Asians than in Europeans, highlighting likely more pivotal roles in Asian patients^2^,^5^. Notably, the PARP value of the significant SNP rs564799 in *IL12A* was almost three times as high in Asians as in Europeans. In contrast, as mentioned in the former part, the effect size of all 10 SNPs, regarding the OR value and direction, were comparable in both Asians and Europeans.

### Systematic annotation and prioritization of the functional SNPs

For the two replicated SNPs, 11 proxy SNPs (r^2^ > 0.8) were extracted, resulting in 13 candidate SNPs for functional annotation. Overall, we found that all of the variants located in non-coding regions of the genome and overlapped with at least one layer of ENCODE data, indicating that these SNPs are likely to influence SLE through mechanisms regulating gene expression. As shown in Table 4, in one context, for the lead SNP rs564799 and its proxies, rs485789 showed the most layers of functional information (i.e., the highest RegulomeDB score + Promoter/Enhancer histone marks and DNAse sites + protein-binding site + matched motifs). This concordance of peaks in rs485789 that correlated disease susceptibility with *IL12A* expression made this SNP a strong candidate as a functional SNP, with *IL12A* as the potentially causal genes. In another context, for the lead SNP rs7726414 and its proxies, rs7726414 intersected with the most layers of functional data (i.e., the highest RegulomeDB score + Promoter/Enhancer histone marks and DNAse sites + protein-binding site + matched motifs) and thus was prioritized as the most likely functional SNP. However, no cis-eQTL effects of rs7726414 and its proxies were identified in the databases applied in the current study.

## Discussion

By investigating the 10 SLE related SNPs in 3 independent East Asian SLE populations, we detected one significant novel loci (*IL12A*) and confirmed one previously reported one (*TCF7*)^8^. With the current replication population, the statistical power for the two significant association signals were 96.8% and 95.7%, respectively. Notably, the locus *IL12B* was identified as novel related genes for SLE in East Asian populations by high-density genotyping^8^, emphasizing the validity and immune relevance of these regions. Moreover, markedly higher RAFs and PARPs for these SNPs were observed in Asian populations compared with Europeans, providing further evidence for a genetic background for the difference in prevalence. The risk alleles and their effects (both effect size and direction) were shared by Asians and Europeans. Consistently with previous studies^2^,^5^, both similarities and differences with respect to RAFs, PARPs and ORs were observed across ethnicities.

Although, the genetic heterogeneity across ancestries would cause different association results. For the 8/10 SNPs for which we did not detect consistent association signals in the current study, different distributions of RAFs and PARPs were also observed. Even using a similar number of cases and controls for both ethnicities, differences in the power to detect significant associations for individuals SNPs across ethnicities appear to depend largely on their allele frequencies. As mentioned above, the detection powers for the 8/10 un-replicated SNPs were about 50–80%. In future work, independent replication in larger populations, especially for the SNPs with lower allele frequencies, will be needed.

More importantly, using the public available databases, we have been able to zoom in on the functional SNPs of the significant SNPs rs564799 and rs7726414, which were proposed to affect the SLE pathology. We found that most of the SLE-related SNPs were located in non-coding regions of the genome and played a role in disease pathogenesis through altering the target gene expression. On one hand, rs485789 in high LD with rs564799 (r^2^ = 1) showed the strongest regulatory evidence among the lead SNP rs564799 and its proxies. It also had a cis-eQTL effect on *IL12A*, indicating rs485789 as the functional SNP and *IL12A* as the potential causal gene. IL12A encodes IL-12α, which is a component of IL-12 (made in B cells, macrophages, dendritic cells and neutrophils). IL-12 is a critical secreted signal in T cell activation. On the other hand, the lead SNP rs7726414 itself was annotated as the strongest regulatory variant. Although no cis-eQTL effects of rs7726414 have been detected in the current study, the annotated gene *TCF7* seemed more likely to be the causal gene. TCF7 is a T cell–specific transcription factor that regulates the expression of CD3. A mouse Tcf7 knockout showed reduced immune-competence of T cells in the periphery. Thus, further fine-mapping analysis and functional studies are still needed to clarify the role of *TCF7* in the pathogenesis of SLE.

In summary, two novel loci reported by SLE GWAS in Europeans have been significantly replicated in three independent East Asian populations. The comparison of RAFs and PARPs in Europeans and Asians provides further evidence for a genetic basis of the high incidence of SLE in Asia compared to Europe. By integrating multiple layers of regulatory information and eQTL mapping, the functional SNPs and genes have been detected.

## Materials and Methods

### Subjects

**Figure 1:**
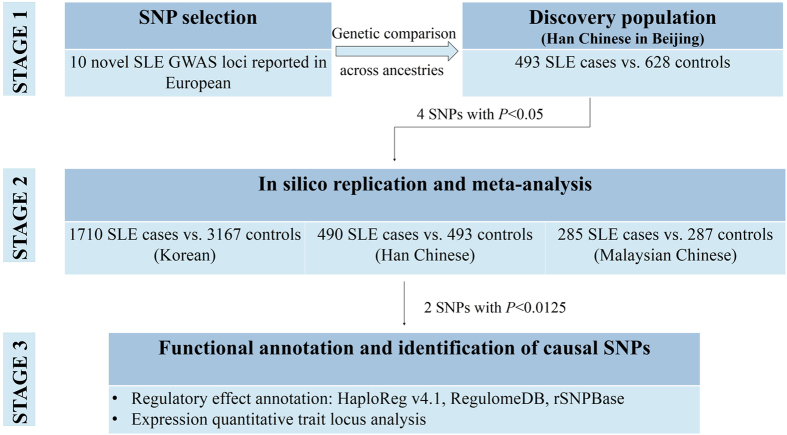
Workflow of our study design. The study was designed in three stages. First, we genotyped the 10 novel genome wide associated loci with European SLE patients in a Han Chinese cohort in Beijing and compared the genetic similarities and differences between the two ancestries. Four out of 10 loci were identified as significant (*P* < 0.1). Second, we performed independent replications of these 4 loci in three cohorts from Korean, Han Chinese and Malaysian Chinese. Consistent associations have been identified in our discovery and replication cohorts for two loci (i.e., *IL12A* and *TCF7*). Third, by integrating different layers of functional data, we identified the most likely functional SNPs for these two loci. Abbreviations: GWAS: genome-wide association study; SLE: systemic lupus erythematosus; SNP: single nucleotide polymorphism.

The current association analysis was conducted in two stages. In the discovery stage, a discovery cohort of Chinese Han ancestry from Northern China was recruited, including 493 SLE cases (age 32.52 ± 12.31 years, female 86.07%) and 628 unrelated healthy controls (age 41.40 ± 11.01 years). In the replication stage, three independent East Asian cohorts, including Koreans, Han Chinese and Malaysian Chinese^8^, were included to validate the associated SNPs (*P* < 0.1). A flowchart of the current study is presented in Fig. 1.

All the patients met the revised SLE criteria of American College of Rheumatology^11^. This investigation was conducted according to the Declaration of Helsinki. The medical ethics committee of Peking University approved the study. All participants gave informed consent.

### SNP selection and genotyping

Although the variant rs7726414 in *TCF7* was also discovered and replicated in our previous study^8^, for consistency and to assess the replication, we evaluated the 10 novel SNPs reported in a recent SLE GWAS conducted in Europeans^7^ without selection. Genotyping was conducted using TaqMan allele discrimination assays as previously reported^12^,^13^,^14^.

To comprehensively evaluate the genetic heterogeneity between Asians and Europeans, we retrieved the summary data of the 10 SNPs from the published SLE GWAS in Europeans^7^. The Han Chinese replication population^8^ were also included for the analysis because both this population and the discovery population were recruited from Northern China. Genotype data for 5 SNPs, including rs6740462, rs564799, rs7726414, rs10774625, and rs9652601, were extracted from the Immunochip, while the remaining 5 SNPs for this cohort were genotyped using TaqMan allele discrimination assays.

### Systematic annotation

**Figure 2:**
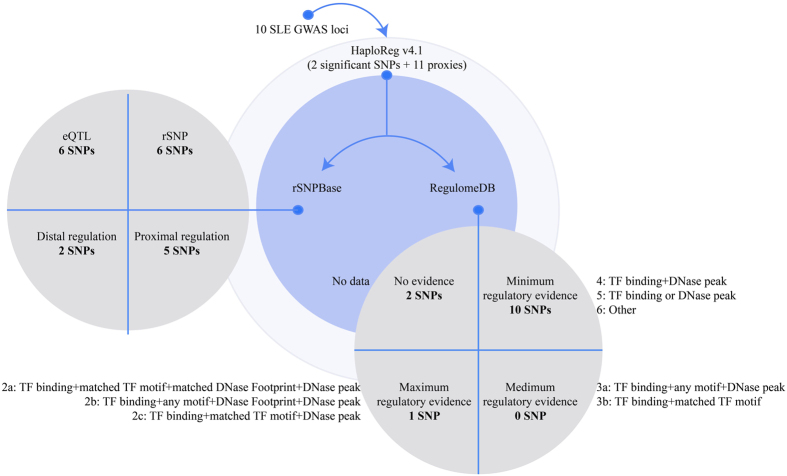
Annotation strategy for the significant variants and their proxies. The figure shows the detailed strategy of our annotation approach used to prioritize functional SNPs. HaploReg v4.1 (http://www.broadinstitute.org/mammals/haploreg/haploreg.php) was used to search proxies (r^2^ ≥ 0.8 in Asian) of the associated SNPs and their binding motifs and epigenetic marks. The rSNPBase (http://rsnp.psych.ac.cn/) database was used to search for regulatory SNPs with experimentally validated regulatory elements controlling transcriptional and post-transcriptional events. RegulomeDB (http://www.regulomedb.org/) was used to search for the regulatory scores of SNPs according to their amount of regulatory information. Abbreviations: eQTL: expression quantitative trait locus; GWAS: genome-wide association study; rSNP: regulatory single nucleotide polymorphism; SLE: systemic lupus erythematosus; SNP: single nucleotide polymorphism.

To prioritize potential functional SNPs and causal genes at the replicated susceptibility loci, we integrated multiple functional data. The detailed procedures of the prioritization process were presented in Fig. 2. Considering that there are often SNPs showing high linkage disequilibrium (LD) with the associated SNPs, we first extracted the proxies (r^2^ > 0.8, 1000 Genomes Project, Asian population as reference) for the significant replicated SNPs using the HaploReg v4.1 database (http://www.broadinstitute.org/mammals/haploreg/haploreg.php), forming the candidate SNPs. The potential functional consequences of the candidate SNPs were predicted using rSNPBase (http://rsnp.psych.ac.cn/) and RegulomeDB databases (http://www.regulomedb.org/). The rSNPBase database provides the regulatory information on SNPs with experimentally validated regulatory elements controlling transcriptional and post-transcriptional events. RegulomeDB ranks SNPs based on the amount of regulatory information with which an SNP intersects. Then, the eQTL mapping data were used to prioritize the replicated SNPs. As a discovery set, the comprehensive and versatile eQTL database seeQTL (http://www.bios.unc.edu/research/genomicsoftware/seeQTL/), which includes various eQTL studies and a meta-analysis of HapMap eQTL information, was investigated, and the results were replicated in lymphoblastoid cell lines of 462 individuals from the 1000 Genomes Project^15^.

### Statistical analysis

Quality control of genotyping, Hardy-Weinberg equilibrium tests, allelic association analyses were performed using PLINK^16^. As a replication, no multiple testing was applied, and *P* < 0.05 was considered significant. ORs and allele frequencies were presented according to the risk alleles identified in Europeans. The contributions of SNPs to the risk of SLE were estimated with PARP, which considers both OR and RAF in the general population, using the formula RAF(OR-1)/[RAF(OR-1) + 1] × 100%^17^. Statistical power was estimated using Power and Sample Size Calculations Version 3.0 (http://biostat.mc.vanderbilt.edu/PowerSampleSize) with a two-sided type I error rate of 0.05.

## Additional Information

**How to cite this article**: Zhang, Y.-m. *et al*. Evaluation of 10 SLE susceptibility loci in Asian populations, which were initially identified in European populations. *Sci. Rep.* **7**, 41399; doi: 10.1038/srep41399 (2017).

**Publisher's note:** Springer Nature remains neutral with regard to jurisdictional claims in published maps and institutional affiliations.

## Figures and Tables

**Table 1:** Associations between the 10 newly mapped loci and susceptibility to systemic lupus erythematosus.

SNP	Risk allele	Chr.	Position (Build 37)	Gene	Discovery population (493 cases vs. 628 controls)		
					RAF (%)	OR	Allele *P*
**rs6740462**	**A**	**2**	**65,667,272**	***SPRED2***	**81.6**/**77.3**	**1.30** (**1.05**–**1.61**)	**1.50** × **10**^**−2**^
rs3768792	G	2	213,871,709	*IKZF2*	20.4/19.1	1.08	0.46
**rs564799**	**C**	**3**	**159,728,987**	***IL12A***	**89.8**/**84.1**	**1.67** (**1.29**–**2.16**)	**9.36** × **10**^**−5**^
**rs7726414**	**T**	**5**	**133,431,834**	***TCF7***	**8.6**/**6.5**	**1.37** (**1.00**–**1.88**)	**5.37** × **10**^**−2**^
rs3794060	C	11	71,187,679	*DHCR7, NADSYN1*	49.0/48.3	1.03	0.77
rs10774625	A	12	111,910,219	*SH2B3*	99.6/99.3	0.62	0.43
rs4902562	A	14	68,731,458	*RAD51B*	59.9/56.8	1.13	0.16
rs9652601	G	16	11,174,365	*CIITA, SOCS1*	76.4/77.2	0.95	0.65
**rs2286672**	**T**	**17**	**4,712,617**	***PLD2***	**41.9**/**37.3**	**1.21** (**1.01**–**1.44**)	**3.51** × **10**^**−2**^
rs887369	C	X	30,577,846	*CXorf21*	97.2/96.7	1.20	0.48

(A) Chr: chromosome; RAF: risk allele frequency; OR: odds ratio; SNP: single nucleotide polymorphism.

(B) 95% confidence interval for OR values are presented only for significant associations in the square brackets.

**Table 2:** Independent replications of associated single nucleotide polymorphisms and meta-analysis.

SNP (risk allele)	Discovery		Independent replication							
	Han Chinese in Beijing (493 cases vs. 628 controls)		Korean (1710 cases vs. 3167 controls)		Han Chinese (490 cases vs. 493 controls)		Malaysian Chinese (285 cases vs. 287 controls)		Meta-analysis	
	RAF% (case/control)	Allele *P*	RAF% (case/control)	Allele *P*	RAF% (case/control)	Allele *P*	RAF% (case/control)	Allele *P*			Adjusted Allele P*	OR
rs6740462 (A)	81.6/77.3	1.50 × 10^−2^	84.0/83.4	0.44	79.3/76.5	0.13	84.9/81.6	0.13	0.12	1.09
**rs564799** (**C**)	**89.8**/**84.1**	**9.36** × **10**^**−5**^	**90.9**/**89.3**	**1.49** × **10**^**−2**^	**87.2**/**85.5**	**0.27**	**87.2**/**82.6**	**2.94** × **10**^**−2**^	**5.91** × **10**^**−4**^	**1.22** (**1.10**–**1.35**)
**rs7726414** (**T**)	**8.6**/**6.5**	**5.37** × **10**^**−2**^	**6.8**/**5.2**	**1.25** × **10**^**−3**^	**8.4**/**4.8**	**1.49** × **10**^**−3**^	**13.0**/**9.6**	**6.58** × **10**^**−2**^	**4.12** × **10**^**−8**^	**1.46** (**1.28**–**1.66**)
rs2286672^*^ (T)	—	—	45.3/46.6	0.24	49.1/47.8	0.56	49.5/51.6	0.48	0.32	0.98

(A) RAF: risk allele frequency; OR: odds ratio; PARP: population-attributable risk percentage; SNP: single nucleotide polymorphism.

(B) ^*^The genotype data of SNP rs2302327 in high linkage disequilibrium with rs2286672 (r^2^ = 0.98 in Asian population) was used for association analysis in Korean, Han Chinese and Malaysian Chinese populations and for the meta-analysis.

(C) 95% confidence interval for OR values are presented only for significant associations in the square brackets.

(D) ^*^The allele *P* was adjusted using the Bonferroni method on 4 SNPs.

**Table 3:** Comparison of risk allele frequencies, odds ratios and population-attributable risk percentages between Chinese and Europeans.

SNP	Risk allele	Gene	Chinese/European controls (1121 vs. 15991)		OR (Chinese/European)	PARP (Chinese/European)
			RAF%	Allele *P*		
rs6740462	A	*SPRED2*	77.0/73.5	9.44 × 10^−3^	1.23/1.20	15.05%/12.82%
rs3768792	G	*IKZF2*	19.2/12.9	2.18 × 10^−9^	1.09/1.26	1.70%/3.25%
**rs564799**	**C**	***IL12A***	**84.7**/**59.3**	**3.27** × **10**^**−64**^	**1.37**/**1.15**	**23.86%**/**8.17%**
**rs7726414**	**T**	***TCF7***	**5.7**/**4.4**	**4.54** × **10**^**−2**^	**1.53**/**1.46**	**2.93%**/**1.98%**
rs3794060	C	*DHCR7, NADSYN1*	49.5/25.6	4.20 × 10^−68^	1.19/1.13	8.60%/3.22%
rs10774625	A	*SH2B3*	99.4/50.1	2.08 × 10^−225^	1.56/1.17	35.76%/7.85%
rs4902562	A	*RAD51B*	58.8/41.7	2.83 × 10^−29^	1.09/1.13	5.03%/5.14%
rs9652601	G	*CIITA, SOCS1*	76.2/66.8	6.96 × 10^−11^	1.04/1.17	2.96%/10.20%
rs2286672	T	*PLD2*	38.1/7.1	1.93 × 10^−266^	1.18/1.24	6.42%/1.68%
rs887369	C	*CXorf21*	97.0/74.7	1.36 × 10^−64^	1.16/1.16	13.43%/10.68%

(A) RAF: risk allele frequency; OR: odds ratio; PARP: population-attributable risk percentage; SNP: single nucleotide polymorphism.

(B) The significant associations were noted in bold.

**Table 4:** Detailed annotation information on the SLE-associated single nucleotide polymorphisms and their proxies.

Lead SNP	Annotated gene	Proxies (r^2^ in ASN)	RegulomeDB score	HaploReg v4.1^1^			rSNPBase		*cis*-eQTL	
				Promoter/Enhancer histone marks and DNAse	Proteins bound	Regulatory motifs	rSNP	Proxy/Distal regulation	seeQTL^2^ (q value)	Selected eQTL hit^3^ (*P* value)
rs564799	IL12A	rs485789 (1)	2b	9	9	4	yes	yes	4.16 × 10^−2^	2.93 × 10^−12^
rs564799	IL12A	rs4680536 (0.96)	4	6	0	2	no	no	4.59 × 10^−2^	7.66 × 10^−13^
rs564799	IL12A	rs485499 (0.97)	4	24	4	0	no	no	—	4.57 × 10^−12^
rs564799	IL12A	rs4679867 (1)	5	3	0	0	yes	yes	—	4.57 × 10^−12^
rs564799	IL12A	rs564799 (1)	5	2	0	0	yes	yes	0.18	2.93 × 10^−12^
rs564799	IL12A	rs564976 (1)	5	3	0	1	yes	yes	—	2.69 × 10^−12^
rs564799	IL12A	rs589446 (1)	6	0	0	4	yes	yes	—	3.09 × 10^−12^
rs564799	IL12A	rs55740284 (0.93)	no evidence	0	0	1	yes	yes	—	—
rs7726414	TCF7	rs4388254 (1)	4	17	0	0	no	no	—	—
rs7726414	TCF7	rs7726414 (1)	4	12	1	4	no	no	—	—
rs7726414	TCF7	rs6874758 (0.98)	5	9	0	5	no	no	—	—
rs7726414	TCF7	rs10077437 (0.98)	6	1	0	2	no	no	—	—
rs7726414	TCF7	rs10463912 (0.89)	no evidence	0	0	5	no	no	—	—

(A) ASN: Asian; eQTL: expression quantitative locus; rSNP: regulatory single nucleotide polymorphism; SLE: systemic lupus erythematosus; SNP: single nucleotide polymorphism.

(B) ^1^The numbers indicate cell types with experimentally validated regulatory elements. ^2^SeeQTL is a comprehensive and versatile eQTL database, including various eQTL studies and a meta-analysis of HapMap eQTL information (http://www.bios.unc.edu/research/genomic_software/seeQTL/). ^3^The selected eQTL hit in this study was driven by the analysis results of mRNA from lymphoblastoid cell lines of 462 individuals from the 1000 Genomes Project (PMID: 24037378).
